# Crystal structure of di­aqua­bis­(4-cyano­pyridine-κ*N*)bis­(thio­cyanato-κ*N*)iron(II) 4-cyano­pyridine disolvate

**DOI:** 10.1107/S205698901700322X

**Published:** 2017-03-03

**Authors:** Aleksej Jochim, Inke Jess, Christian Näther

**Affiliations:** a Institut für Anorganische Chemie, Christian-Albrechts-Universität Kiel, Max-Eyth Strasse 2, D-24118 Kiel, Germany

**Keywords:** crystal structure, hydrogen bonding, 4-cyano­pyridine, iron, thio­cyanate

## Abstract

The asymmetric unit of the title compound, [Fe(NCS)_2_(C_6_H_4_N_2_)_2_(H_2_O)_2_]·2C_6_H_4_N_2_, comprises one Fe^II^ cation occupying an inversion centre as well as one thio­cyanate anion, one water mol­ecule and two 4-cyano­pyridine mol­ecules in general positions. The iron cations are coordinated by two N-bonded thiocyanate anions, two (pyridine)N-bonded 4-cyano­pyridine ligands and two water mol­ecules into discrete complexes. The resulting coordination polyhedron can be described as a slightly distorted octa­hedron. The discrete complexes are connected through centrosymmetric pairs of (pyridine)C—H⋯N(cyano) hydrogen bonds into chains that are further linked into a three-dimensional network through inter­molecular O—H⋯N hydrogen bonds involving the 4-cyano­pyridine solvent mol­ecules.

## Abstract 2

The crystal structure of the title compound consists of discrete octa­hedral complexes and additional cyano­pyridine solvate mol­ecules that are linked by inter­molecular O—H⋯N and C—H⋯N hydrogen bonding into a three-dimensional network.

## Chemical context   

Thio­cyanate anions are versatile ligands that can coordinate in different modes to metal cations. In most cases the anionic ligands are terminally N-bonded to the metal cation but there are also several examples for a *μ-*_1,3_ bridging mode (Werner *et al.*, 2015 ▸; Boeckmann & Näther, 2012 ▸; Palion-Gazda *et al.*, 2015 ▸). The latter coordination is of special inter­est if the compounds contain paramagnetic metal cations because then cooperative magnetic properties can be expected (Palion-Gazda *et al.*, 2015 ▸). In this context, we have reported on several compounds with one- or two-dimensional structures based on Mn, Fe, Co or Ni as metals, thio­cyanate ligands and different N-donor co-ligands that show different magnetic properties (Suckert *et al.*, 2016 ▸; Rams *et al.*, 2017 ▸; Boeckmann *et al.*, 2012 ▸). Whereas compounds with a terminal coordination of the anionic ligands can usually be synthesized straightforwardly, compounds with bridging ligands are sometimes difficult to obtain from solution. Therefore, we have developed an alternative procedure which is based on thermal decomposition of precursors with a terminal NCS coordination that frequently transform into the desired polymeric compounds on heating. In the course of our investigations on the synthesis of coordination polymers with iron as metal, thio­cyanate ligands and 4-cyano­pyridine as co-ligands, we obtained the title compound which was identified by single crystal X-ray diffraction. Unfortunately, all samples were always contaminated with a second unknown crystalline phase, preventing any further investigations.
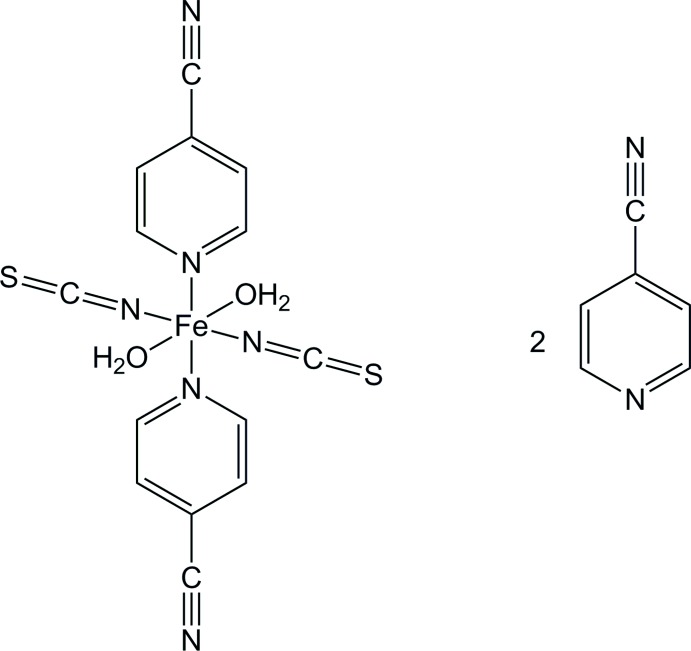


## Structural commentary   

**Figure 1:**
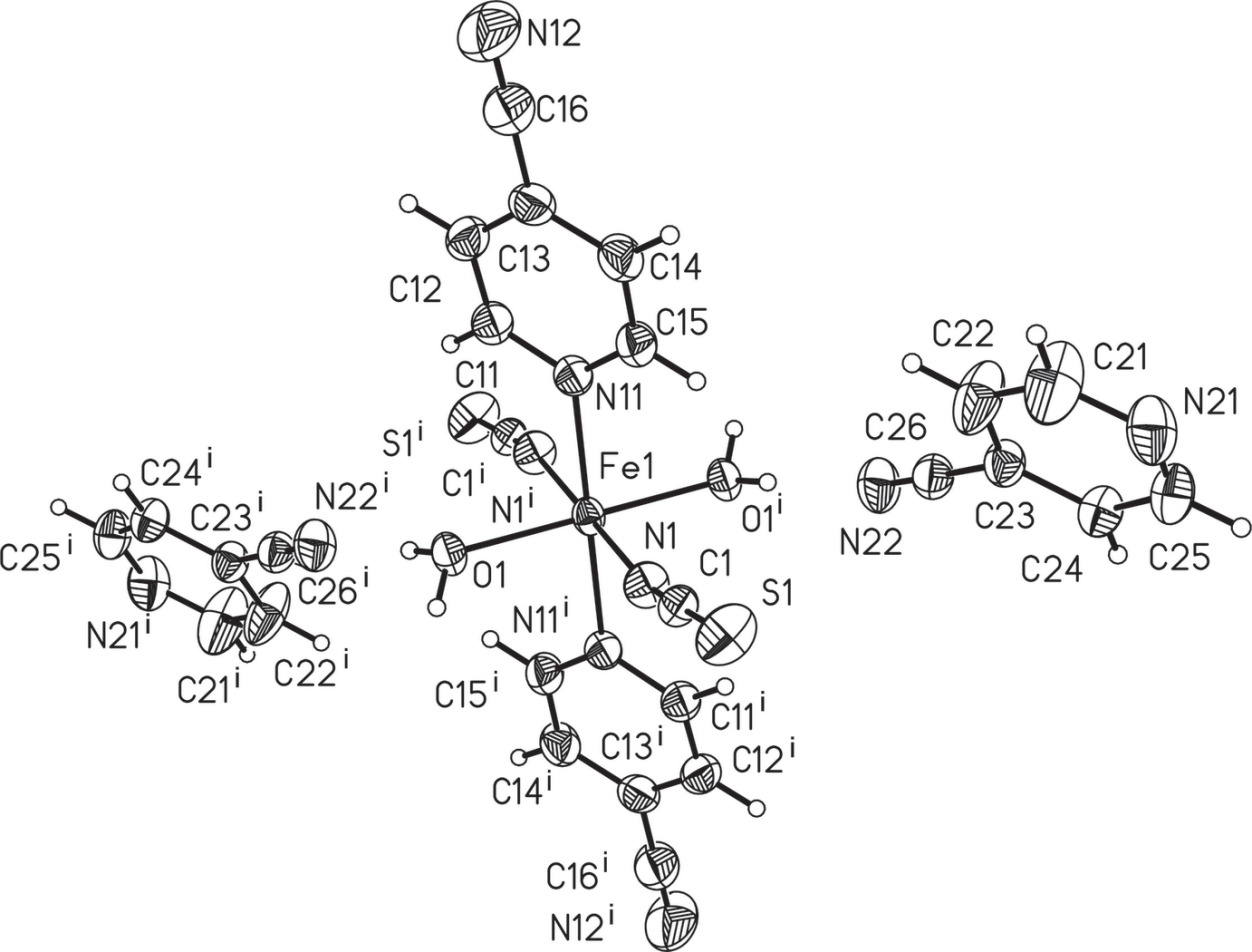
The discrete complex and the solvent mol­ecule of the title compound with labeling and displacement ellipsoids drawn at the 50% probability level. [Symmetry code: (i) 1 − *x*, 1 − *y*, 2 − *z*.]

The asymmetric unit of [Fe(NCS)_2_(C_6_H_4_N_2_)_2_(H_2_O)_2_]·2C_6_H_4_N_2_ contains one Fe^II^ cation that is located on an inversion centre, one thio­cyanate anion, one water mol­ecule and two 4-cyano­pyridine mol­ecules (Fig. 1 ▸). Discrete centrosymmetric [Fe(NCS)_2_(C_6_H_4_N_2_)_2_(H_2_O)_2_] complexes are formed, in which the Fe^II^ cations are octa­hedrally coordinated by two N-bonded thio­cyanate anions, two (pyridine)N-bonded 4-cyano­pyridine ligands and two water mol­ecules, each of them in a *trans*-position (Fig. 1 ▸). The disparate bond lengths are similar to those in related thio­cyanate compounds. The distortion of the octa­hedron is also reflected by the deviation of the bond angles from ideal values. The structure contains additional 4-cyano­pyridine solvate mol­ecules that are located in the cavities of the structure.

## Supra­molecular features   

**Figure 2:**
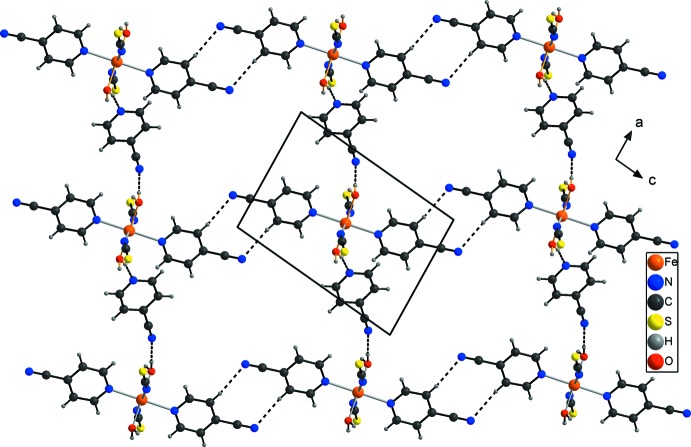
Part of the crystal structure of the title compound in a view along the *b* axis with emphasis on the connection of discrete complexes and solvent mol­ecules by inter­molecular hydrogen bonding (dashed lines).

**Figure 3:**
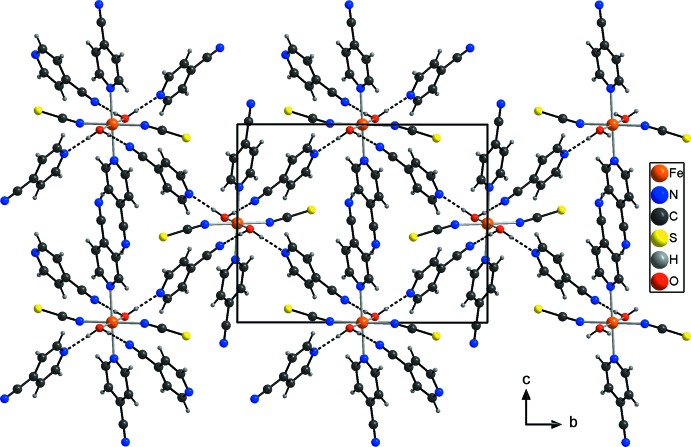
The crystal structure of the title compound in a view along the *a* axis. Inter­molecular hydrogen bonding is shown as dashed lines.

The discrete complexes are linked into chains parallel to [101] by centrosymmetric pairs of inter­molecular C—H⋯N hydrogen bonds between the cyano group of the coordinating 4-cyano­pyridine ligand and one of the pyridine H atoms (Fig. 2 ▸, Table 1 ▸). These chains are further linked by the 4-cyano­pyridine solvate mol­ecules through inter­molecular O—H⋯N hydrogen bonding. One water H atom is hydrogen-bonded to the N atom of the cyano group and the other H atom to the pyridine N atom of another 4-cyano­pyridine solvate mol­ecule. Since all water H atoms are involved in hydrogen bonding, each of the complexes is surrounded by four 4-cyano­pyridine ligands, of which two are hydrogen-bonded *via* the cyano group, whereas the other two are hydrogen-bonded *via* the pyridine N atom (Fig. 3 ▸, Table 1 ▸). This arrangement leads to a three-dimensional network structure. It is noted that there are additional short contacts between the thio­cyanate anions and the pyridine H atoms of the coordinating 4-cyano­pyridine ligand of a neighbouring complex, which is indicative of weak C—H⋯S hydrogen bonding (Table 1 ▸).

## Database survey   

In the Cambridge Structure Database (Version 5.38, last update 2016; Groom *et al.*, 2016 ▸), five structures of coordination polymers with 4-cyano­pyridine and thio­cyanate as ligands are reported, in which the metal cations are solely connected through *μ*-_1,3_ bridging thio­cyanate anions. Two of these compounds contain copper, two cadmium and one is a bi­metal­lic compound in which copper and mercury are present. The two copper-containing compounds are built up of chains, in which the cations are either tetra­hedrally (Lin *et al.*, 2004 ▸) or octa­hedrally (Machura *et al.*, 2013*a* ▸) coordinated. In the bimetallic compound the cations are linked into a three-dimensional structure (Machura *et al.*, 2013*b* ▸), whereas the two cadmium-containing compounds exhibit either one-dimensional or three-dimensional coordination networks (Chen *et al.*, 2002 ▸).

## Synthesis and crystallization   

Iron(II) chloride tetra­hydrate, potassium thio­cyanate and 4-cyano­pyridine were obtained from Alfa Aesar and used without further purification.

29.8 mg iron(II) chloride tetra­hydrate (0.15 mmol) and 29.2 mg KSCN (0.30 mmol) were reacted with 62.5 mg 4-cyano­pyridine (0.60 mmol) in 1.5 ml water at room temperature. After two days, single crystals suitable for structure analysis were obtained. The batch contained a small amount of an additional crystalline phase that could not be identified.

## Refinement   

Crystal data, data collection and structure refinement details are summarized in Table 2 ▸. Hydrogen atoms of the water mol­ecule were located from a difference map, and C-bound hydrogen atoms were refined in calculated positions [C—H = 0.95 Å and O—H = 0.84 Å] with *U*_iso_(H) = 1.2*U*_eq_(C) [1.5 for *U*_eq_(O)] using a riding model (O—H hydrogen atoms were allowed to rotate but not to tip).

## Supplementary Material

Crystal structure: contains datablock(s) I. DOI: 10.1107/S205698901700322X/wm5371sup1.cif

Structure factors: contains datablock(s) I. DOI: 10.1107/S205698901700322X/wm5371Isup2.hkl

CCDC reference: 1534965

Additional supporting information:  crystallographic information; 3D view; checkCIF report

## Figures and Tables

**Table 1:** Hydrogen-bond geometry (Å, °)

*D*—H⋯*A*	*D*—H	H⋯*A*	*D*⋯*A*	*D*—H⋯*A*
C12—H12⋯N12^i^	0.95	2.52	3.437 (3)	162
C14—H14⋯S1^ii^	0.95	3.01	3.960 (2)	177
O1—H1⋯N22^iii^	0.84	2.00	2.8380 (19)	177
O1—H2⋯N21^iv^	0.84	1.89	2.7159 (19)	168

Symmetry codes: (i) 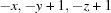; (ii) 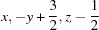; (iii) 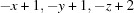; (iv) 
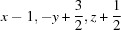
.

**Table 2:** Experimental details

Crystal data	
Chemical formula	[Fe(NCS)_2_(C_6_H_4_N_2_)_2_(H_2_O)_2_]·2C_6_H_4_N_2_
*M*_r_	624.49
Crystal system, space group	Monoclinic, *P*2_1_/*c*
Temperature (K)	200
*a*, *b*, *c* (Å)	8.5376 (4), 15.220 (1), 12.1214 (6)
β (°)	96.195 (6)
*V* (Å^3^)	1565.88 (15)
*Z*	2
Radiation type	Mo *K*α
μ (mm^−1^)	0.66
Crystal size (mm)	0.13 × 0.10 × 0.06

Data collection	
Diffractometer	Stoe IPDS1
Absorption correction	Numerical (*X-RED* and *X-SHAPE*; Stoe & Cie, 2008 ▸)
*T*_min_, *T*_max_	0.884, 0.953
No. of measured, independent and observed [*I* > 2σ(*I*)] reflections	18486, 3743, 2960
*R*_int_	0.047
(sin θ/λ)_max_ (Å^−1^)	0.663

Refinement	
*R*[*F* ^2^ > 2σ(*F* ^2^)], *wR*(*F* ^2^), *S*	0.037, 0.094, 1.03
No. of reflections	3743
No. of parameters	188
H-atom treatment	H-atom parameters constrained
Δρ_max_, Δρ_min_ (e Å^−3^)	0.26, −0.46

Computer programs: *X-AREA* (Stoe & Cie, 2008 ▸), *SHELXS97* and *XP* (Sheldrick, 2008 ▸), *SHELXL2014* (Sheldrick, 2015 ▸), *DIAMOND* (Brandenburg, 2014 ▸) and *publCIF* (Westrip, 2010 ▸).

## supplementary crystallographic information

### Crystal data

**Table:**

[Fe(NCS)_2_(C_6_H_4_N_2_)_2_(H_2_O)_2_]·2C_6_H_4_N_2_	*F*(000) = 640
*M**_r_* = 624.49	*D*_x_ = 1.324 Mg m^−^^3^
Monoclinic, *P*2_1_/*c*	Mo *K*α radiation, λ = 0.71073 Å
*a* = 8.5376 (4) Å	Cell parameters from 18864 reflections
*b* = 15.220 (1) Å	θ = 3.8–56.3°
*c* = 12.1214 (6) Å	µ = 0.66 mm^−^^1^
β = 96.195 (6)°	*T* = 200 K
*V* = 1565.88 (15) Å^3^	Block, yellow
*Z* = 2	0.13 × 0.10 × 0.06 mm

### Data collection

**Table:**

Stoe IPDS-1 diffractometer	2960 reflections with *I* > 2σ(*I*)
Phi scans	*R*_int_ = 0.047
Absorption correction: numerical (*X-RED* and *X-SHAPE*; Stoe & Cie, 2008)	θ_max_ = 28.1°, θ_min_ = 2.7°
*T*_min_ = 0.884, *T*_max_ = 0.953	*h* = −11→10
18486 measured reflections	*k* = −20→20
3743 independent reflections	*l* = −16→16

### Refinement

**Table:**

Refinement on *F*^2^	Hydrogen site location: mixed
Least-squares matrix: full	H-atom parameters constrained
*R*[*F*^2^ > 2σ(*F*^2^)] = 0.037	*w* = 1/[σ^2^(*F*_o_^2^) + (0.0581*P*)^2^ + 0.1102*P*] where *P* = (*F*_o_^2^ + 2*F*_c_^2^)/3
*wR*(*F*^2^) = 0.094	(Δ/σ)_max_ = 0.001
*S* = 1.03	Δρ_max_ = 0.26 e Å^−^^3^
3743 reflections	Δρ_min_ = −0.46 e Å^−^^3^
188 parameters	Extinction correction: SHELXL2014 (Sheldrick, 2015), Fc^*^=kFc[1+0.001xFc^2^λ^3^/sin(2θ)]^-1/4^
0 restraints	Extinction coefficient: 0.019 (3)

### Special details

**Table:**

Geometry. All esds (except the esd in the dihedral angle between two l.s. planes) are estimated using the full covariance matrix. The cell esds are taken into account individually in the estimation of esds in distances, angles and torsion angles; correlations between esds in cell parameters are only used when they are defined by crystal symmetry. An approximate (isotropic) treatment of cell esds is used for estimating esds involving l.s. planes.

### Fractional atomic coordinates and isotropic or equivalent isotropic displacement parameters (Å^2^)

**Table:**

	*x*	*y*	*z*	*U*_iso_*/*U*_eq_	
Fe1	0.5000	0.5000	1.0000	0.02513 (11)	
N1	0.58788 (18)	0.62971 (9)	0.99472 (12)	0.0358 (3)	
C1	0.62673 (19)	0.69914 (10)	0.96961 (13)	0.0305 (3)	
S1	0.68128 (7)	0.79604 (3)	0.93149 (5)	0.05165 (16)	
N11	0.42359 (17)	0.50738 (9)	0.81712 (11)	0.0317 (3)	
C11	0.2830 (2)	0.47429 (11)	0.77885 (14)	0.0351 (4)	
H11	0.2267	0.4414	0.8283	0.042*	
C12	0.2158 (2)	0.48550 (11)	0.67119 (15)	0.0390 (4)	
H12	0.1150	0.4617	0.6472	0.047*	
C13	0.2990 (2)	0.53234 (13)	0.59914 (14)	0.0399 (4)	
C14	0.4458 (2)	0.56588 (13)	0.63609 (15)	0.0425 (4)	
H14	0.5055	0.5976	0.5877	0.051*	
C15	0.5029 (2)	0.55172 (12)	0.74578 (14)	0.0381 (4)	
H15	0.6036	0.5747	0.7718	0.046*	
C16	0.2332 (3)	0.54478 (18)	0.48511 (18)	0.0570 (6)	
N12	0.1795 (3)	0.5537 (2)	0.39540 (17)	0.0837 (8)	
O1	0.28045 (13)	0.55033 (7)	1.02877 (9)	0.0320 (2)	
H1	0.2201	0.5134	1.0535	0.048*	
H2	0.2728	0.5951	1.0684	0.048*	
N21	1.2086 (2)	0.80713 (12)	0.64837 (15)	0.0523 (4)	
C21	1.0626 (3)	0.7796 (2)	0.6202 (2)	0.0774 (9)	
H21	1.0072	0.8031	0.5546	0.093*	
C22	0.9863 (3)	0.71950 (19)	0.67912 (19)	0.0685 (8)	
H22	0.8813	0.7018	0.6554	0.082*	
C23	1.0672 (2)	0.68573 (11)	0.77413 (14)	0.0347 (4)	
C24	1.2197 (2)	0.71283 (11)	0.80597 (15)	0.0378 (4)	
H24	1.2775	0.6904	0.8713	0.045*	
C25	1.2856 (2)	0.77362 (13)	0.73992 (17)	0.0445 (4)	
H25	1.3907	0.7923	0.7609	0.053*	
C26	0.9913 (2)	0.62137 (12)	0.83758 (14)	0.0361 (4)	
N22	0.9307 (2)	0.56987 (11)	0.88643 (14)	0.0460 (4)	

### Atomic displacement parameters (Å^2^)

**Table:**

	*U*^11^	*U*^22^	*U*^33^	*U*^12^	*U*^13^	*U*^23^
Fe1	0.02351 (17)	0.02120 (16)	0.03167 (17)	−0.00047 (11)	0.00745 (11)	0.00057 (11)
N1	0.0402 (9)	0.0231 (6)	0.0448 (8)	−0.0057 (5)	0.0075 (6)	0.0016 (5)
C1	0.0277 (8)	0.0303 (8)	0.0329 (8)	−0.0006 (6)	0.0004 (6)	−0.0007 (6)
S1	0.0599 (3)	0.0304 (2)	0.0628 (3)	−0.0137 (2)	−0.0017 (2)	0.0132 (2)
N11	0.0305 (7)	0.0324 (7)	0.0328 (7)	0.0002 (5)	0.0053 (5)	−0.0015 (5)
C11	0.0343 (9)	0.0308 (8)	0.0408 (9)	−0.0028 (6)	0.0061 (7)	−0.0035 (6)
C12	0.0341 (9)	0.0375 (9)	0.0446 (9)	0.0010 (7)	0.0001 (7)	−0.0080 (7)
C13	0.0397 (10)	0.0458 (10)	0.0338 (8)	0.0094 (8)	0.0022 (7)	−0.0055 (7)
C14	0.0393 (10)	0.0550 (11)	0.0342 (8)	0.0011 (8)	0.0086 (7)	0.0046 (8)
C15	0.0325 (9)	0.0472 (10)	0.0353 (8)	−0.0032 (7)	0.0071 (7)	0.0014 (7)
C16	0.0451 (12)	0.0819 (16)	0.0433 (11)	0.0035 (11)	0.0015 (9)	−0.0003 (10)
N12	0.0618 (14)	0.140 (2)	0.0460 (11)	−0.0095 (14)	−0.0079 (9)	0.0126 (13)
O1	0.0272 (6)	0.0284 (5)	0.0422 (6)	−0.0004 (4)	0.0117 (5)	−0.0031 (4)
N21	0.0464 (10)	0.0518 (10)	0.0607 (10)	−0.0051 (8)	0.0144 (8)	0.0215 (8)
C21	0.0541 (15)	0.111 (2)	0.0643 (15)	−0.0162 (14)	−0.0084 (11)	0.0537 (15)
C22	0.0417 (12)	0.105 (2)	0.0554 (13)	−0.0231 (12)	−0.0112 (10)	0.0401 (13)
C23	0.0313 (9)	0.0379 (9)	0.0351 (8)	−0.0039 (7)	0.0048 (6)	0.0051 (6)
C24	0.0338 (10)	0.0367 (9)	0.0421 (9)	−0.0014 (7)	0.0005 (7)	0.0066 (7)
C25	0.0341 (10)	0.0406 (10)	0.0593 (11)	−0.0058 (7)	0.0068 (8)	0.0074 (8)
C26	0.0315 (9)	0.0419 (9)	0.0351 (8)	−0.0026 (7)	0.0039 (6)	0.0020 (7)
N22	0.0397 (9)	0.0490 (9)	0.0508 (9)	−0.0068 (7)	0.0122 (7)	0.0104 (7)

### Geometric parameters (Å, º)

**Table:**

Fe1—O1^i^	2.0888 (11)	C14—H14	0.9500
Fe1—O1	2.0888 (11)	C15—H15	0.9500
Fe1—N1	2.1153 (13)	C16—N12	1.141 (3)
Fe1—N1^i^	2.1153 (13)	O1—H1	0.8400
Fe1—N11^i^	2.2451 (14)	O1—H2	0.8400
Fe1—N11	2.2451 (14)	N21—C21	1.325 (3)
N1—C1	1.158 (2)	N21—C25	1.329 (3)
C1—S1	1.6286 (16)	C21—C22	1.368 (3)
N11—C15	1.337 (2)	C21—H21	0.9500
N11—C11	1.338 (2)	C22—C23	1.377 (3)
C11—C12	1.378 (3)	C22—H22	0.9500
C11—H11	0.9500	C23—C24	1.380 (3)
C12—C13	1.382 (3)	C23—C26	1.442 (2)
C12—H12	0.9500	C24—C25	1.382 (3)
C13—C14	1.382 (3)	C24—H24	0.9500
C13—C16	1.447 (3)	C25—H25	0.9500
C14—C15	1.383 (3)	C26—N22	1.140 (2)
			
O1^i^—Fe1—O1	180.0	C14—C13—C16	120.42 (19)
O1^i^—Fe1—N1	90.55 (5)	C13—C14—C15	117.86 (17)
O1—Fe1—N1	89.45 (5)	C13—C14—H14	121.1
O1^i^—Fe1—N1^i^	89.45 (5)	C15—C14—H14	121.1
O1—Fe1—N1^i^	90.55 (5)	N11—C15—C14	123.35 (17)
N1—Fe1—N1^i^	180.0	N11—C15—H15	118.3
O1^i^—Fe1—N11^i^	88.63 (5)	C14—C15—H15	118.3
O1—Fe1—N11^i^	91.37 (5)	N12—C16—C13	179.0 (3)
N1—Fe1—N11^i^	90.59 (5)	Fe1—O1—H1	114.2
N1^i^—Fe1—N11^i^	89.41 (5)	Fe1—O1—H2	121.3
O1^i^—Fe1—N11	91.37 (5)	H1—O1—H2	104.5
O1—Fe1—N11	88.63 (5)	C21—N21—C25	117.42 (17)
N1—Fe1—N11	89.41 (5)	N21—C21—C22	124.4 (2)
N1^i^—Fe1—N11	90.59 (5)	N21—C21—H21	117.8
N11^i^—Fe1—N11	180.0	C22—C21—H21	117.8
C1—N1—Fe1	166.44 (14)	C21—C22—C23	117.5 (2)
N1—C1—S1	178.74 (15)	C21—C22—H22	121.2
C15—N11—C11	117.64 (15)	C23—C22—H22	121.2
C15—N11—Fe1	123.39 (12)	C22—C23—C24	119.69 (17)
C11—N11—Fe1	118.54 (11)	C22—C23—C26	119.08 (17)
N11—C11—C12	123.23 (17)	C24—C23—C26	121.22 (15)
N11—C11—H11	118.4	C23—C24—C25	117.94 (17)
C12—C11—H11	118.4	C23—C24—H24	121.0
C11—C12—C13	118.18 (17)	C25—C24—H24	121.0
C11—C12—H12	120.9	N21—C25—C24	123.03 (18)
C13—C12—H12	120.9	N21—C25—H25	118.5
C12—C13—C14	119.72 (17)	C24—C25—H25	118.5
C12—C13—C16	119.85 (19)	N22—C26—C23	179.1 (2)

Symmetry code: (i) −*x*+1, −*y*+1, −*z*+2.

### Hydrogen-bond geometry (Å, º)

**Table:**

*D*—H···*A*	*D*—H	H···*A*	*D*···*A*	*D*—H···*A*
C12—H12···N12^ii^	0.95	2.52	3.437 (3)	162
C14—H14···S1^iii^	0.95	3.01	3.960 (2)	177
O1—H1···N22^i^	0.84	2.00	2.8380 (19)	177
O1—H2···N21^iv^	0.84	1.89	2.7159 (19)	168

Symmetry codes: (i) −*x*+1, −*y*+1, −*z*+2; (ii) −*x*, −*y*+1, −*z*+1; (iii) *x*, −*y*+3/2, *z*−1/2; (iv) *x*−1, −*y*+3/2, *z*+1/2.
